# Novel facile method for obtaining CdSe/polyaniline/C_60_ composite materials

**DOI:** 10.1038/srep32237

**Published:** 2016-08-30

**Authors:** Edina Rusen, Aurel Diacon, Alexandra Mocanu, Leona Cristina Nistor

**Affiliations:** 1 University Politehnica of Bucharest, Department of Bioresources and Polymer Science, 1- 7 Gh. Polizu Street, 011061 Bucharest, Romania; 2 National Institute of Materials Physics, 105 bis Atomistilor, 077125 Magurele-Ilfov, Romania

## Abstract

This study presents a novel method for the oxidative polymerization of aniline (ANI) by employing fullerene C_60_/cadmium selenide (CdSe) quantum dots, as promoting agent of the polymerization system. The polymerization initiation mechanism is based on the difference between the HOMO-LUMO energy levels of the components which permits the formation of a continuous donor-acceptor exchange. Both the polymerization reaction evolution and the molecular weights of the obtained polymers have been characterized. The novelty of the paper consists in the synthesis of a novel nano-composite material through a novel polymerization technique. The resulting material containing PANI, CdSe quantum dots and C_60_ has been characterized by UV-Vis, NIR, fluorescence, TEM and GPC analyses.

Polyaniline (PANI) is one of the oldest known conductive polymers^1^. Due to its low cost, good environmental stability and adequate electrical properties^2^,^3^, PANI has found application in the fabrication of bio/chemical sensors^4^,^5^, solar cells^6^, organic light emitting diodes^7^, supercapacitors^8^, field effect transistors^9^ and electromagnetic interference shielding^10^. The synthesis and doping methods of PANI have a crucial role in determining the properties of the obtained materials^3^,^11^.

Polyaniline (PANI) can be synthesized by electrochemical methods (potentiostatically or galvanostatically), by oxidation of the monomer using inert electrodes, such as stainless steel, platinum, gold, different types of carbon (vitreous or pyrolytic graphite), and glass covered with metal oxides. PANI can also be chemically synthesized in acidic media using an oxidizing agent, such as potassium dichromate, ammonium persulfate, hydrogen peroxide, cerium nitrate, etc.

Over the past few decades hybrid nano-composites containing inorganic nanoparticles or carbon materials have become particularly attractive because of their promising applications in electronic and optoelectronic industries^3^,^12^,^13^,^14^,^15^.

Thus, the aim of this study consist in a new synthesis route of ANI polymerization using strongly electrophile molecule, e.g. fullerene C_60_ and quantum dots nanoparticles of CdSe (3 nm) as electron donating species. Considering the energy levels of the HOMO and LUMO for the two molecules, ANI and C_60_, the oxidative polymerization of the monomer is difficult to be achieved. In this case, a promotor for the polymerization is necessary, presenting electron donating properties towards ANI, making more accessible the electron transfer to C_60_. Quantum dots nanoparticles of CdSe (3 nm) were chosen as the promoting component.

This straightforward synthesis approach allows the facile manufacturing of a novel hybrid material with improved properties. Therefore, both the materials as well as the synthesis strategy are original.

## Materials

Aniline (ANI) (Merck) has been purified through vacuum distillation and kept on molecular sieves 4 Å. Fullerenes (C_60_) (Aldrich) has been used as received. Dimethylformamide (DMF) (Aldrich), toluene (T) (Fluka) and methanol (Aldrich) have been used without further purification.

## Methods

CdSe-quantum dots (3 nm) have been prepared according to our paper^16^.

*Polymerization procedure*: in 5 mL of CdSe solution (0.04 mol/L in DMF), 2 mL ANI and 2 mL C_60_ in T (1% weight) were added at room temperature under stirring for 2 hours. Methanol has been used as precipitation medium of the polymers. The polymers were filtered, washed several times with methanol and dried until a constant mass was attained.

### Characterization

The UV-Vis-NIR spectra were recorded using a spectrofotometer Shimadzu UV-3600. The fluorescence spectra were registered using a Jasco FP-6500 Able Jasco spectrofluorimeter.

The molecular weights of the resulted polymers and oligomers were analyzed using PL-GPC 50 Integrated GPC/SEC System (Agilent Technologies) using a 1 mL/min THF flow rate and a column oven temperature of 50 °C.

Infrared absorption spectra have been recorded at room temperature with a Nicolet 6700 FTIR spectrometer in the range of 4000–400 cm^−1^.

The elemental analysis was performed with Perkin Elmer 2400 Series II CHNS/O Analyzer equipped with thermal conductivity detector (combustion temperature 975 °C, reduction temperature 500 °C).

The high resolution transmission electron microscopy (HRTEM) studies were performed on an atomic resolution analytical JEOL JEM-ARM 200F electron microscope, operating at 200 kV. Specimens for HRTEM were prepared in the following way: a drop of the solution was put on holey carbon TEM grid and dried at 100 °C for 5 min.

## Results and Discussion

Literature presents the capacity of ANI to polymerize through an oxidative mechanism, respectively “pseudo-living cationic” polymerization process^15^,^17^. The first step of polymerization consists in the monomer oxidation. Therefore, the monomer polymerization could be attained by an electron transfer process to a strongly electrophile molecule, e.g. fullerene C_60_^18^, followed by chain growth process. Considering the energy levels of the HOMO and LUMO for the two molecules, ANI and C_60_, the oxidation of the monomer is difficult to be achieved. In this case, a promotor for the polymerization is necessary, presenting electron donating properties towards ANI, making more accessible the electron transfer to C_60._ Quantum dots nanoparticles of CdSe (3 nm) were chosen as the promoting component. Thus, energy levels of the components involved in the process are as follows: **ANI** HOMO = −8.10 eV, LUMO = 1.44 eV^19^, **C**_**60**_ HOMO = −6.1 eV, LUMO = −4.3 eV^20^, **CdSe** HOMO = −5.6 eV, LUMO = −3.4−eV^21^.

In order to highlight the ANI polymerization process, UV-Vis and fluorescence analyses were performed at different intervals.

**Figure 1:**
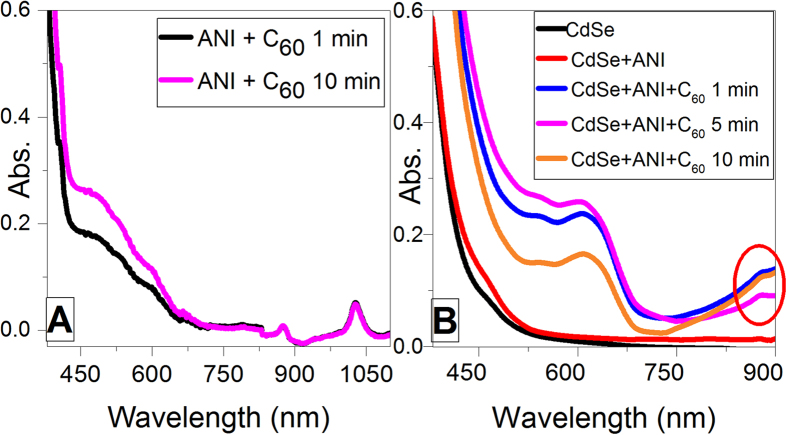
(**A**) UV-Vis spectra for ANI-C_60_ charge transfer complex formation (**B**) UV-Vis spectra at 50 °C and reaction intervals.

In Fig. 1A is presented the absorption spectra of ANI/C_60_ mixture which confirms the formation of a charge transfer complex^18^. The analysis of Fig. 1B reveals the initial absorption specific for the CdSe quantum dots at 470 nm. There are no modifications in the visible range of the spectra after the addition of the ANI, until introduction of C_60_, which leads to the formation of the peaks at 620 nm and 890 nm. The first signal is specific for the PANI structure in basic medium^22^, the absorbance declining with the reaction progress and pH decrease, evidence for monomer consumption. The signal at 890 nm is specific for the fullerene radical carbocation^23^.

**Figure 2:**
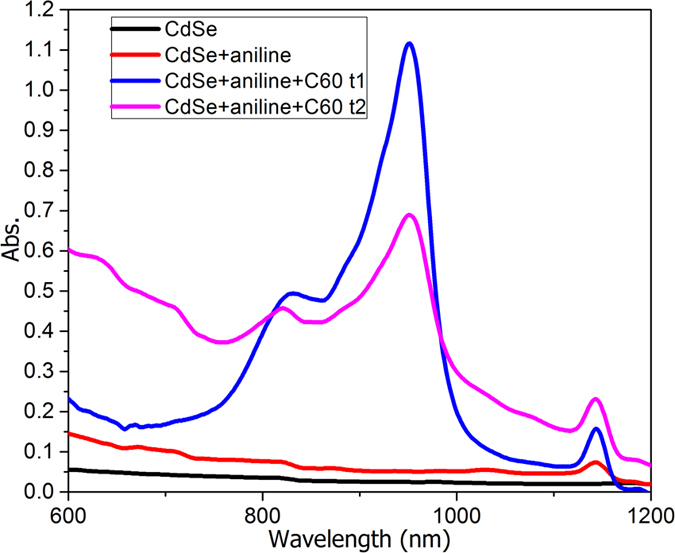
NIR spectra at different intervals.

In order to highlight the formation of the intermediate species during the polymerization process, Vis-NIR spectroscopy was used to study the evolution of the system for a wavelength range between 700 nm and 1200 nm (Fig. 2). The signals at 820 nm and 950 nm are specific to the carbocation resulting from C_60_^23^, whereas the 1144 nm peak sustains the oxidized species of ANI. The signal at 1144 nm appears also for the CdSe-ANI mixture without C_60_, confirming this designation.

**Figure 3:**
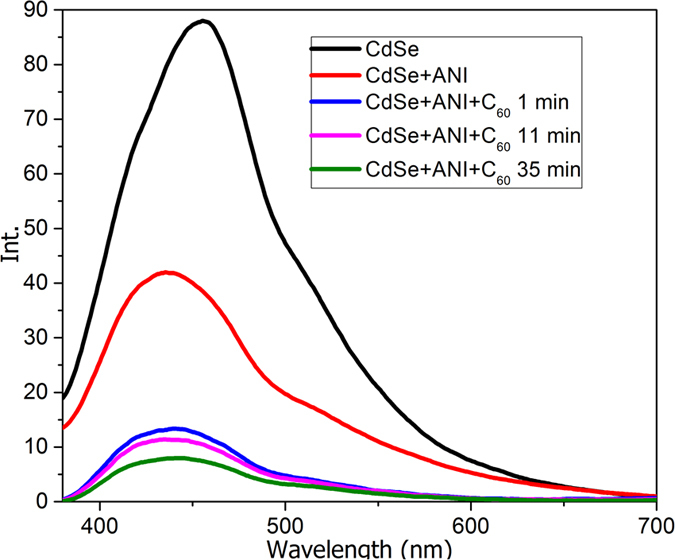
Emission spectra at an excitation wavelength of 370 nm.

The fluorescence spectra (Fig. 3) reveal that the emission of CdSe (max around 460 nm) decreases on addition of ANI followed by a further decrease upon introduction of C_60_. This aspect sustains the donor activity of CdSe towards both components. The emission intensity registers only small decrease in time after initial quench caused by C_60_ insertion in the system. This stabilization and the lack of emission increase indicate the hypothesis that the CdSe quantum dots act only as promotor for the polymerization.

**Figure 4:**
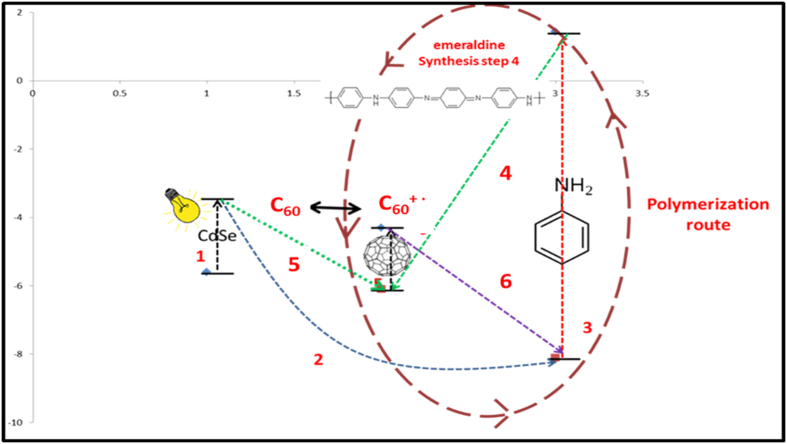
Schematic representation of the oxidative polymerization of ANI using CdSe-C_60_ system.

Based on the presented experimental data, the schematic representation of the polymerization process (including the HOMO-LUMO levels for each component) is presented in Fig. 4.

The polymerization process stages are: 1. excitation of the CdSe quantum-dots; 2. electron donating process from CdSe^*^ to ANI molecules; 3. excitation of ANI molecules; 4. electron transfer from ANI^*^ to C_60_, accompanied by the polymerization process; 5. C_60_ excitation; 6. electron transfer from C_60_ to ANI with the formation of fullerene carbocation (C_60_^+ •^); 7. electron transfer from ANI^*^ to C_60_^+ •^ accompanied by the polymerization process.

**Figure 5:**
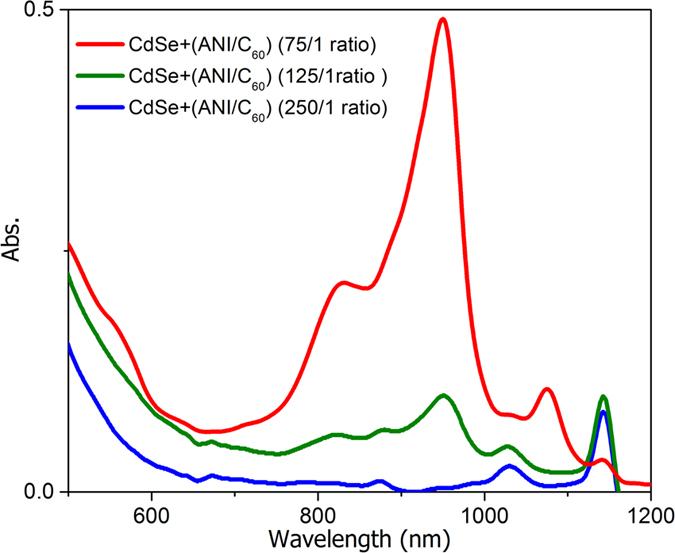
NIR at different ANI/C_60_ molar ratio.

Following the polymerization experiments, we have determined that the CdSe quantum dots have a promoter role and do not actively participate in the polymerization process. Based on the steps 4 and 6 presented in Fig. 4, it is possible that the ratio between ANI and C_60_ to influence the polymerization rate by modifying the concentration of the formed intermediate species. Thus, the process was followed by Vis-NIR spectroscopy, at different molar ratios of ANI/C_60_ (Fig. 5).

It is evident that with the acceptor concentration increase, the absorbance specific for the radical carbocation, as well as the absorbance of PANI formed also increases.

In order to better characterize the reaction mechanism two directions were followed: 1) polymerization experiments at different CdSe concentrations; 2) experiments at different ANI/C_60_ ratio. In the first case, the values for the conversion are low, decreasing with the concentration increase of CdSe in the system; thus, confirming its promotor role in the polymerization process.

Therefore, our next step was the study of the polymerization process kinetic at different concentrations of C_60_.

**Figure 6:**
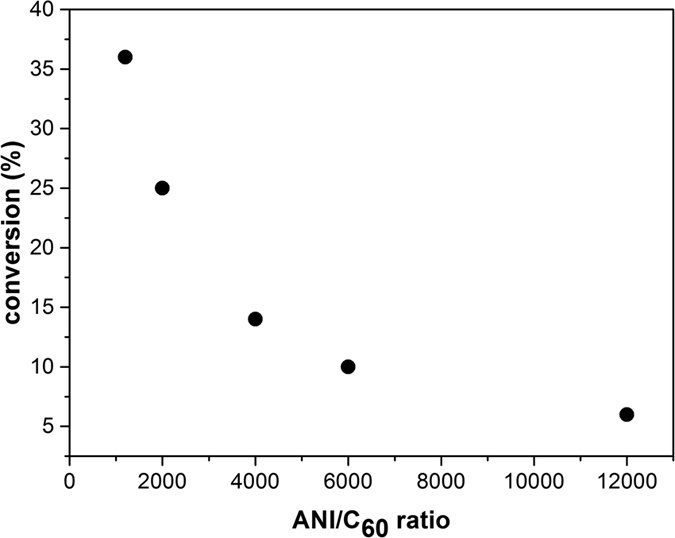
The conversion versus ANI/C_60_ ratio.

In Fig. 6, it is presented the dependence of the conversion on the C_60_ concentration. It can be observed that the conversion value decreases with the ANI/C_60_ ratio increase.

In order to confirm a “living” polymerization mechanism GPC analyses were performed on the obtained polymers. The GPC results for lowest and highest conversion are summarized in Table 1.

From the GPC results it can be concluded that the conversion increase is accompanied by an increase of the molecular weight, respectively a decrease of the polydispersity index.

**Figure 7:**
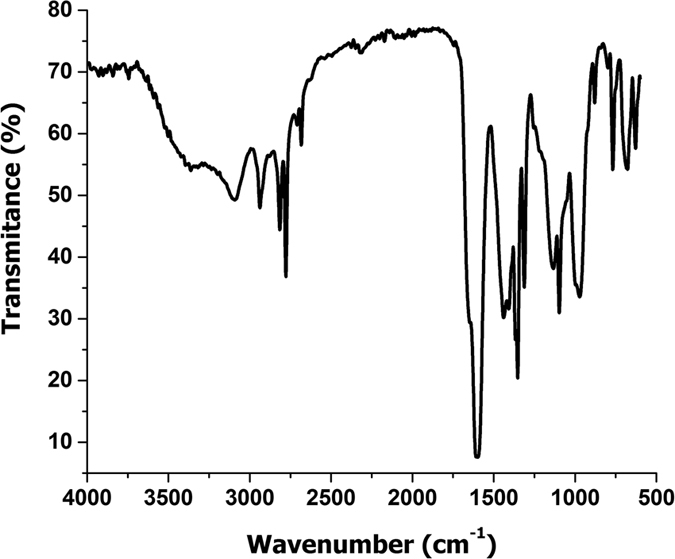
FTIR spectra for PANI synthesized using CdSe/C_60_ system.

In order to confirm the PANI structure, we have performed FTIR analysis (Fig. 7). The characteristics bands that can be noticed in the FTIR spectra are: 1598, 1439, 1220 and 1132 cm^−1^ due to quinoid ring C=C stretching, benzenoid C-C stretching, C-N stretching band and C-N^+•^ 24 stretching vibration. The presence of C_60_ can be confirmed by the presence of specific peaks at 1435 and 1186 cm^−1^ 14.

Elemental analysis of the PANI sample revealed the following values: 77.3% C, 7% H and 15.7% N. The results sustain the polymer structure of PANI.

**Figure 8:**
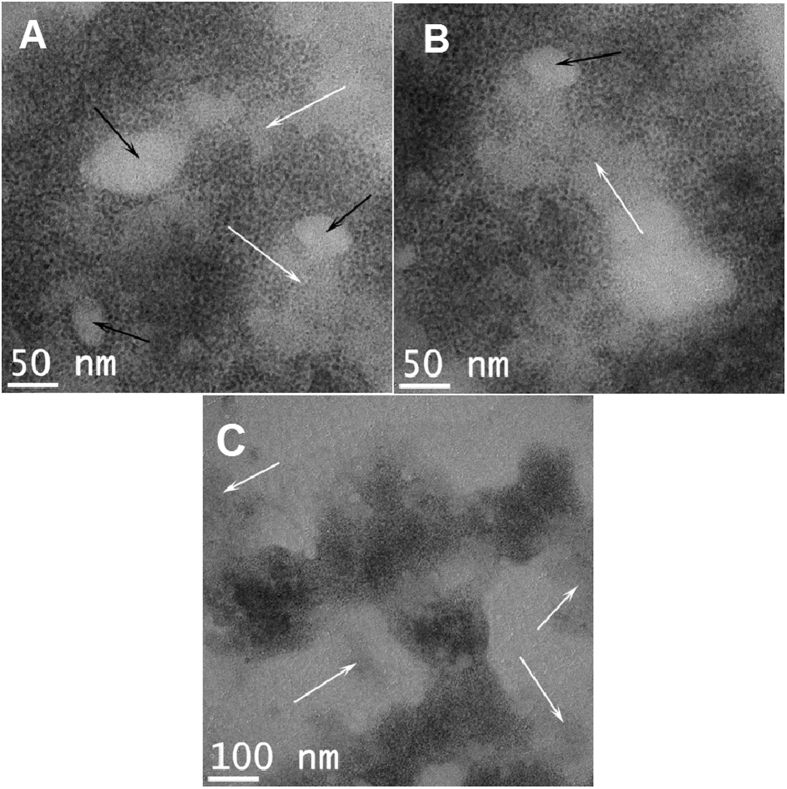
*HRTEM images* of the morphology of aniline layer covered with CdSe nanoparticles. White arrows indicate almost bare aniline.

For the characterization of the hybrid material in view of future applications, HRTEM analysis was performed (Fig. 8).

The CdSe nanoparticles (dark contrast) is not uniformly spread over the PANI layer (gray contrast). White arrows indicate almost bare regions on the PANI layer where the density of CdSe nanoparticles is very small. Black arrows show holes in the aniline layer were the carbon grid is revealed (light gray contrast). The CdSe nanoparticles are in close contact with the PANI layer. No CdSe particles were observed outside PANI layer, this can be observed in the TEM image below taken at an even lower magnification (Fig. 7C). C_60_ crystallites were not observed.

## Conclusions

This study presents a novel method for the oxidative polymerization of ANI by employing C_60_/(CdSe) quantum dots, as promoting agent of the polymerization system. The polymerization initiation mechanism is based on the difference between the HOMO-LUMO energy levels of the components which permit the formation of a continuous donor-acceptor exchange. In order to highlight the formation of the intermediate species during the polymerization process, UV-Vis and Vis-NIR spectroscopy was used. Thus, the fullerene carbocation radical has been put into evidence.

Polymerization experiments performed at different ANI/C_60_ ratio and CdSe concentrations revealed a decreasing conversion with the increase of CdSe. Further, GPC analyses confirmed a ‘’living” mechanism of the polymerization.

The HRTEM investigation of the morphology revealed CdSe nanoparticles non-uniformly distributed inside the PANI layer, however no free quantum dots were observed.

## Additional Information

**How to cite this article**: Rusen, E. *et al*. Novel facile method for obtaining Cdse/polyaniline/C_60_ composite materials. *Sci. Rep*. **6**, 32237; doi: 10.1038/srep32237 (2016).

## Figures and Tables

**Table 1:** GPC results.

Conversion (%)	Mn (g/mol)	Mw (g/mol)	PDI
5.1	1757	2025	1.15
37.5	11804	12395	1.06
